# Pilot Study on the Geographical Mapping of Genetic Diversity among European Chestnut (*Castanea sativa* Mill.) Cultivars in Southern Italy

**DOI:** 10.3390/plants12040917

**Published:** 2023-02-17

**Authors:** Marina Maura Calandrelli, Angelina Nunziata, Luigi De Masi

**Affiliations:** 1 Institute of Research on Terrestrial Ecosystems (IRET), National Research Council (CNR), Via P. Castellino 111, 80131 Napoli, Italy; 2 Research Centre for Olive, Fruit and Citrus Crops, Council for Agricultural Research and Economics (CREA), Via Torrino 2, 81100 Caserta, Italy; 3 Institute of Biosciences and BioResources (IBBR), National Research Council (CNR), Via Università 133, Portici, 80055 Napoli, Italy

**Keywords:** agrobiodiversity, genetic distance, DNA analysis, Geographic Information System (GIS), landscape genetics

## Abstract

Knowledge of the spatial distribution of European chestnut (*Castanea sativa* Mill.) cultivar diversity is essential for managing and conserving the genetic resources of this fruit tree species in Southern Italy. To this goal, the present work investigated the feasibility of mapping, through spatial representation, the distribution of genetic diversity of traditional chestnut varieties in the area of the Roccamonfina Regional Park in the Campania Region. After Principal Coordinates Analysis (PCoA) of molecular-genetic data, chestnuts formed varietal groups in a leopard spot on PCoA plots with a relatively high degree of genetic diversity. Successively, a Geographic Information System (GIS) tool utilized these molecular-genetic data to create a genetic divergence surface by geospatial interpolation on the geographic map of the Regional Park corresponding to each chestnut variety. The regions containing more biodiversity richness resulted in differentially colored from those containing cultivars less genetically distant from each other; thus, the area in study was consistently colored according to the allelic richness as evaluated by molecular-genetic markers. The combined use of tools for molecular and spatial analysis allowed for drafting genetic landscapes with the aim of extracting useful information for the safeguarding of the chestnut biodiversity at risk.

## 1. Introduction

The European or sweet chestnut (*Castanea sativa* Mill.) is a tall tree species, belonging to the Fagaceae family, of considerable agroforestry importance worldwide [1] and, more recently, of nutraceutical interest [2,3,4]. Sweet chestnut trees are spread throughout the European continent as natural forests, partially exploited as coppice, as well as specialized orchards for nut production. Natural forests are composed of many different individuals, sharing a certain number of local alleles, localized on the highest and steepest parts of restricted and isolated areas in the inland mountains. Chestnut orchards, instead, occupy wider areas in which one or few genotype(s) are clonally spread on different rootstocks. These cloned individuals, coming from nearby or from very distant areas, can modify the allelic composition of the natural forests they surround thanks to natural pollen flow [5,6]. The complex equilibrium between natural forests and anthropically influenced orchards needs to be quantified and described to policymakers for informed biodiversity management. The geographical distribution and the varietal composition of naturalized chestnut forests are the results of combined action over time of natural processes and human activities; however, nowadays, we are experiencing a biodiversity reduction due to both natural and anthropic factors [7,8,9]. In this respect, it is important to highlight that the positive effects of ecosystem services provided by forests are considered essential to contribute to the maintenance of human health [10].

Since 2005, the accidental entry into the Campania Region (Southern Italy) of the invasive alien species Hymenoptera: Cynipidae *Dryocosmus kuriphilus* Yasumatsu, 1951 (Chinese chestnut gall wasp) has been threatening the survival of traditional chestnut varieties, especially those not very represented on the regional territory [11,12]. At the same time, the plague of the chestnut gall wasp drastically reduced the production of marketable fruits, bringing chestnut cultivation to its knees [9,12]. This ecological contingency is putting at risk a unique heritage consisting of cultivars from Campania of high value and with a long local tradition, also protected by national and European marks. However, the susceptibility to gall wasps has been shown to depend on specific chestnut genotypes [13,14,15]. In this context, a deep knowledge of the territorial mapping of chestnut genetic diversity is crucial to plan strategies for managing and conservation of these precious genetic resources.

Landscape genetics would allow a correct evaluation of the geographic patterns of genetic diversity using reliable molecular tools together with spatial statistics. The routine identification of chestnut cultivars is mainly based on morphological traits, which are unreliable indicators of the specific chestnut genotype as they can be influenced by environmental conditions. Recent research has investigated the distribution of genetic diversity of the chestnut on a large scale at the European level [16,17]. However, to our best knowledge, in Campania, there are no studies at the local level that could have a more practical value, although Italy was indicated as the European area at a higher priority for chestnut genetic conservation [16,17]. In the past, Italy was the main European producer of chestnut fruits (30%), with about 50% of the Campania Region nationwide [18], where chestnut currently still plays an important economic role [11] with a cultivation area of 13,800 ha followed by other Italian regions [19]. In this region, the territory of the Regional Park “Volcanic Area of Roccamonfina and Foce Garigliano” has an ancient and strong chestnut vocation with a considerable concentration of chestnut biodiversity represented by the main typical varieties ‘Lucente’, ‘Marzatica’, ‘Mercogliana’, ‘Napoletana’, ‘Olefarella’, ‘Paccuta’, ‘Tempestiva’, and ‘San Pietro’. Among these, ‘Tempestiva’, ‘Mercogliana’, and ‘Napoletana’ have a good chance of guaranteeing an interesting economic return due to their early market entry and superior organoleptic characteristics. Furthermore, ‘Tempestiva’ and ‘Paccuta’ can boast the national recognition of Traditional Agri-food Products (P.A.T.) [20]. More recently, this area was recognized as a specific place of origin for the European Union Protected Geographical Indication “Castagna di Roccamonfina” (EUOJ L 285/3, 7 November 2022) [21].

For all these reasons, in the current study, a survey of molecular-genetic tools and Geographic Information System (GIS) applications available for mapping the distribution of chestnut biodiversity on the territory of Roccamonfina Regional Park was carried out. We verified the possibility of combining previously acquired molecular data on the genetic diversity of cultivated chestnuts [1] with spatial statistics by GIS technologies in order to map them locally. In this work, the genetic spatial analysis of chestnut trees, representative of the eight most interesting and renowned typical cultivars present in the Roccamonfina area, was performed by contextually elaborating distinct datasets derived from analyses performed by different molecular markers. Thus, this investigation represented a pilot study on the feasibility of mapping the genetic diversity on the territory through geospatial representation, involving the chestnut samples genetically analyzed in our previous work [1]. In this way, genetic diversity maps of chestnut cultivars based on different classes of molecular markers were produced, which could be considered a prototype to utilize locally for the management and conservation of these precious genetic resources directly in their growing areas.

## 2. Results and Discussion

It is notoriously recognized that Campania Region is an area with a strong tradition of chestnut cultivation, where chestnuts are present in very diverse environmental and edaphoclimatic conditions that contribute to the formation of specialized and selected chestnut orchards [1,9,22]. In this regard, the present study has created a mapping prototype of the genetic diversity distribution of traditional chestnut varieties through molecular tools and GIS applications in the Regional Park “Volcanic Area of Roccamonfina and Foce Garigliano” with the aim of extracting useful information for the management and conservation of chestnut biodiversity at risk.

### 2.1. Molecular-Genetic Data Analysis of Chestnut Genetic Resources

**Figure 1:**
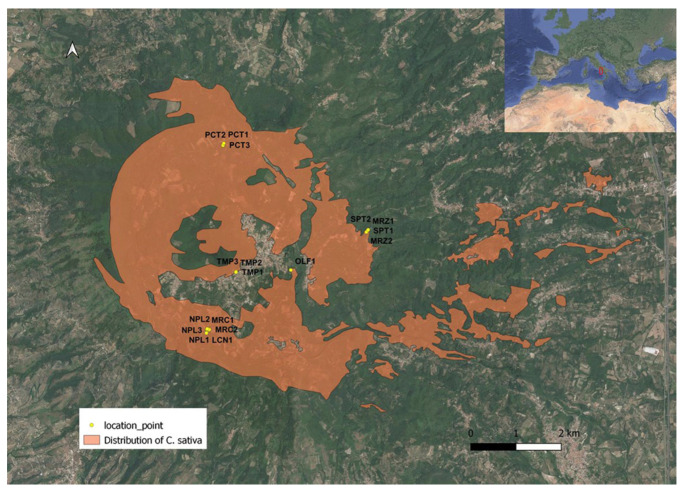
Location of sampling points of *C. sativa* and distribution of chestnut trees in the study area within the Regional Park “Volcanic Area of Roccamonfina and Foce Garigliano”, Campania (Italy).

DNA-based tools were previously tested on the main chestnut varietal genotypes cultivated and appreciated for the quality of their fruits in the protected area of Roccamonfina in Campania Region [1], i.e., ‘Lucente’ (LCN), ‘Marzatica’ (MRZ), ‘Mercogliana’ (MRC), ‘Napoletana’ (NPL), ‘Olefarella’ (OLF), ‘Paccuta’ (PCT), ‘San Pietro’ (SPT), and ‘Tempestiva’ (TMP). Seventeen chestnut trees, belonging to these eight renowned cultivars, were geographically located (Figure 1) and genetically investigated both by Random Amplified Polymorphic DNA (RAPD) and Kompetitive Allele-Specific PCR (KASP) molecular markers.

**Figure 2:**
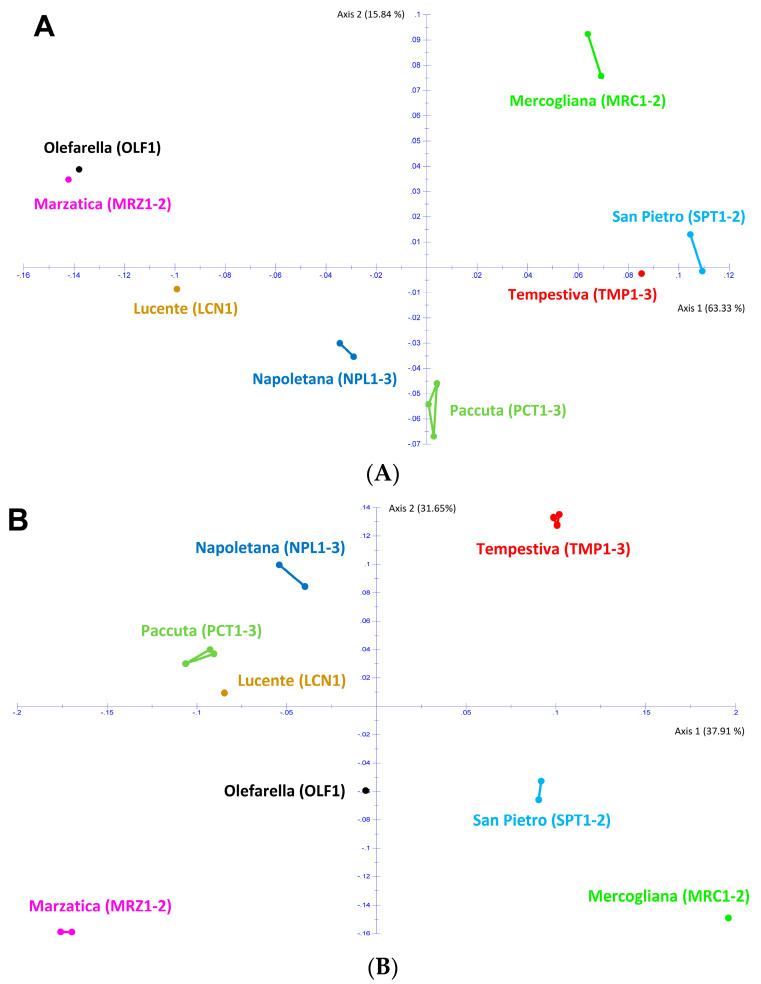
PCoA of the 17 chestnut samples from eight *C. sativa* cultivars of the Regional Park “Volcanic Area of Roccamonfina and Foce Garigliano” based on their DNA polymorphisms obtained by RAPD (**A**) and KASP (**B**) molecular markers.

The genetic diversity values were pairwise calculated for the 17 chestnut samples and then elaborated by PCoA for RAPD and KASP markers, respectively. Both PCoA evidenced the genetic distribution of the analyzed genotypes on the first two axes, explaining the 79% of the variability detected by RAPD markers (Figure 2A) and the 70% of the variability detected by KASP markers (Figure 2B).

Both panels coherently evidenced that some pairs of cultivars are more similar, such as ‘Napoletana’/‘Paccuta’ or ‘Mercogliana’/‘San Pietro’, while other cultivar pairs are more distant, such as ‘Marzatica’ and ‘Tempestiva’. However, there are some differences peculiar for each molecular marker. In particular, of all these cultivars in Figure 2A, the RAPD results closely clustered together ‘Marzatica’ and ‘Olefarella’, such as ‘San Pietro’ and ‘Tempestiva’ (albeit these are farther apart), so forming heterogeneous groups. The other cultivars occupied their own distinct position, forming monovarietal groups apart. The KASP results, reported in Figure 2B, showed ‘Lucente’ and ‘Paccuta’ clustered together, while all the other cultivars formed homogeneous groups. Altogether, most of the 17 chestnut samples coming from the same area were separated by a relatively high degree of genetic divergence, forming varietal groups distributed in a leopard spot on both PCoA plots. The analysis of these data confirmed that it is possible to distinguish at the DNA level the chestnut trees belonging to the renowned cultivars of the Campania [1].

The two molecular methods allowed us to have a raw estimation of the genetic distances existing among the analyzed cultivars in order to test the GIS application. In particular, as RAPD markers were obtained via PCR amplification on random regions of chestnut genomic DNA by using several arbitrary primers, they include much information on the existing genetic variability, resulting in specific patterns of amplification [1]. KASP analysis is based on the detection of SNP that have the highest level of resolution among molecular markers; however, each SNP is usually a bi-allelic type of marker. KASP data are highly reliable and gave realistic values highlighting the absence of genetic variability among clones of the same cultivar [1]. This is particularly evident in the PCoA of Figure 2B, where samples of the same cultivar mostly overlap with each other, and the little detected distances are due to not available data. Nonetheless, the number of monitored SNP in this pilot study is restricted to 37, so the distances between pairs of cultivars are not perfectly detected. The use of at least 120 SNP *loci* could give the same amount of information obtained using about 12 highly polymorphic markers as RAPD [1].

### 2.2. Geospatial Mapping of Genetic Diversity among Chestnut Cultivars

**Figure 3:**
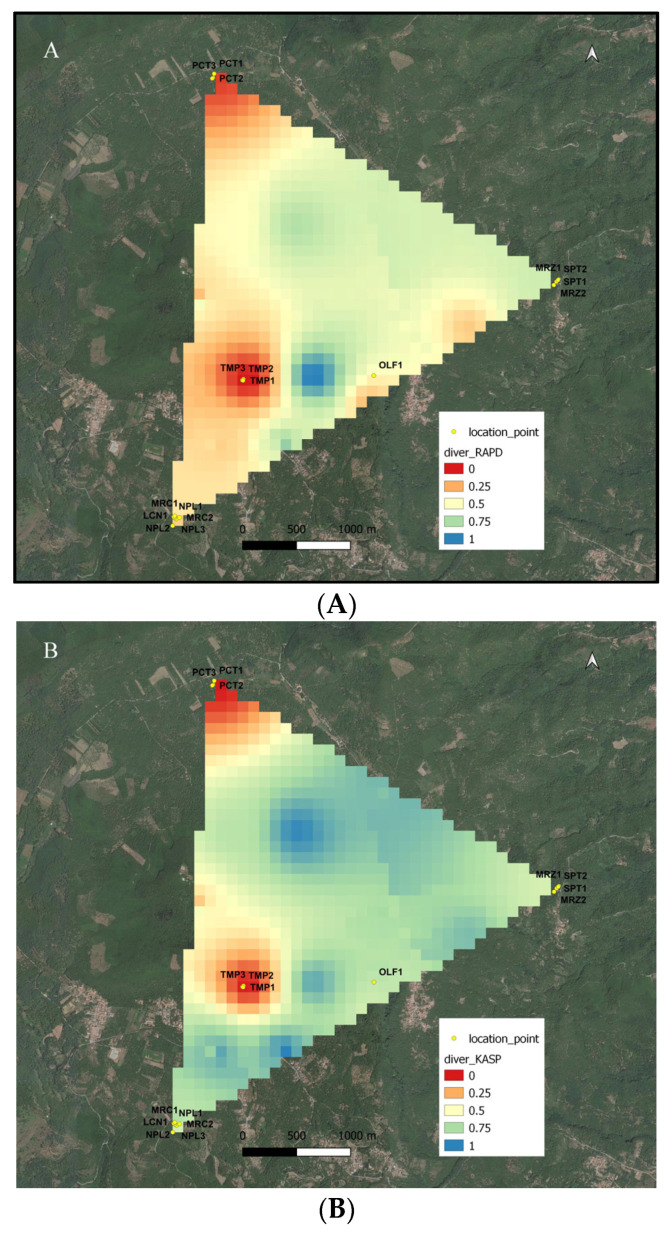
Distribution of the genetic distances based on RAPD (**A**) and KASP (**B**) molecular markers in the geographic space of the study area.

The GIS analysis allowed visualizing the distributions of the genetic distance RAPD (Figure 3A) and KASP (Figure 3B) in the geographic space of the Regional Park, correlating them to the position of each chestnut tree. The GIS tool created a genetic divergence surface based on pairwise genetic diversity in the single population for each type of molecular marker. The geospatial interpolation produced distribution data of the genetic diversity of the chestnut on the geographic map of the Regional Park and allowed to optimize the visualization and interpretation of molecular-genetic data.

Nevertheless, the potential and the limits of this method of analysis for mapping the chestnut agro-biodiversity of a territory are particularly evident in the two maps of landscape genetics shown in Figure 3A,B. This allows us explaining how much a careful sampling of representative individuals is essential for an informative map. As shown, the number and the distances among sampled individuals are essential for the final coloring of the color-coded map. As an example, in each of the two regions colored in red, three trees belonging to each of the ‘Tempestiva’ and ‘Paccuta’ cultivars were sampled. Depending on the geographical distances of the trees of the same cultivar, and between these trees and the nearest ones of the other cultivar, the red area surrounding ‘Paccuta’ trees is wider than that surrounding ‘Tempestiva’ trees. Instead, the area surrounding ‘Olefarella’, which also contains only one cultivar, is colored pale orange and is narrow, depending on the fact that one tree was sampled and is not so far from other different trees. On the other hand, in the same map, two regions containing more than one cultivar are colored in cold colors, indicating more biodiversity richness. The coldest one includes two trees of ‘San Pietro’ and two trees of ‘Marzatica’, which are very distant from each other in the corresponding PCoA representations in Figure 2. The other area, despite including more cultivars and trees (three for ‘Napoletana’, two for ‘Mercogliana’, and one for ‘Lucente’), results in slightly warmer colors, depending on the fact that these three cultivars are less genetically distant from each other (see PCoA of Figure 2); thus, the detected allelic richness of the coldest area is effectively greater than that of the warmest area. Besides, differences between Figure 3A,B underline the specific potential of the two adopted molecular technologies for estimating genetic distances. Altogether, the two maps of landscape genetics describe a very similar situation, underlining the robustness of this kind of mapping, independently from the adopted molecular tool. Nonetheless, more contrast between hot and cold zones is evidenced in Figure 3B (for KASP) than in 3A (for RAPD). This fact is related to a narrower clustering of individuals from the same cultivar, as shown in Figure 2B (for KASP). In a denser sampling, the difference among the used molecular techniques could be more evident, imposing a more accurate evaluation of real genetic distances. In fact, it was estimated that SNP-based technologies, such as KASP, would be really informative, provided that at least 120 assays are included in the analyses [1]. However, sampling at higher population density will be necessary to acquire more detailed information on the spatial distribution of genetic diversity of chestnut cultivars.

Similarly, by combining microsatellite markers and geo-statistical methods, other research identified Italy as a hotspot of natural genetic diversity in large-scale European studies on wild chestnut forests [16,17]. Since Italy was highlighted for conservation priority, small-scale studies by integrating genetic and spatial data on chestnut orchards are essential for mapping varietal diversity that could have an important practical impact on improving chestnut cultivation. After validation on a sample of larger size with the support of different molecular markers, the use of the new set of SNP *loci* should be useful for large-scale genetic analysis at the regional level in this economically important fruit species. Consequently, these innovative genetic-spatial tools will allow us to better evaluate the landscape through models of genetic diversity targeted to the conservation, protection, and management of chestnut varieties traditionally cultivated in the Campania Region that are currently at high risk of genetic erosion.

## 3. Materials and Methods

### 3.1. Plant Material and Molecular Marker Analysis

In this study, we utilized chestnut sampling carried out in our previous work by Nunziata et al. (2020) [1] from the most renowned typical cultivars of *C. sativa* (Table 1) located at the Regional Park “Volcanic Area of Roccamonfina and Foce Garigliano” of Campania Region (Italy). These chestnut cultivars have been previously verified by phenological, agronomic, and carpological traits using International Union for the Protection of New Varieties of Plants (UPOV; https://www.upov.int/portal/index.html.en, accessed on 15 June 2022) descriptors by Nunziata et al. (2020) [1]. The different number of samples for each cultivar is related to its abundance or rarity on the territory. All sampled trees were georeferenced and photographed.

DNA profiling of the chestnut cultivars by RAPD analysis was previously performed by Nunziata et al. (2020) [1]. We used the results of RAPD molecular markers published in our previous research [1]. RAPD alleles for each primer were scored as the number of bands per genotype and coded in binary format for presence or absence. The RAPD allelic table was used to calculate the genetic distances according to Dice’s coefficient by DARwin v.6.0.021 software [23], and the resulting triangular dissimilarity matrix was used for Principal Coordinates Analysis (PCoA) representation.

In our previous work, 37 SNPs were selected for developing KASP assays designed for molecular discrimination of chestnut cultivars, and analyses were previously conducted by Nunziata et al. (2020) [1]. We used the results of KASP molecular markers published in our previous article [1]. In brief, the results were analyzed by the allelic discrimination tool of the Bio-Rad CFX Manager Software v. 3.1 and exported after automatic allele calling. The KASP allelic table was used for analysis using the DARwin v.6.0.021 software [23]. Genetic distances were computed by the simple matching index for diploid codominant markers and the resulting triangular dissimilarity matrix was used for PCoA representation.

### 3.2. Geographical Mapping of Chestnut Genetic Diversity

GIS is a powerful tool for spatial analysis, data visualization, and mapping [24]. In this study, the Genetic Landscapes GIS Toolbox, developed by Perry et al. (2010) [25], was used for the geospatial analysis performed by ESRI ArcGIS 9.3 software [Environmental Systems Research Institute, Inc., 2010, Redlands, CA, USA]. Several standalone software tools allow the identification of genetic diversity. However, the development of these tools within a GIS framework is much more advantageous because, using existing GIS data, they produce outputs compatible with other GIS. This tool creates genetic landscape surfaces directly from tables containing pairwise genetic distance and sample location coordinates, beyond a grid file representing the extent of the analysis area [26]. This method reduces the complexity of constructing and analyzing raster surfaces. The single species diversity tool makes within-site population genetic diversity analysis for multiple populations or collection points within a species. The inverse distance weighted (IDW) spatial interpolation algorithm is used to generate a surface from the mapped genetic distance values. IDW interpolation uses a linearly weighted combination of a series of sample points that determine cell values. It is based on the assumption that the interpolating surface is influenced most by nearby points and less by more distant points. Therefore, an output raster surface with color-coded values, scaled between 0 and 1, has been created for each molecular marker dataset.

## 4. Conclusions and Perspectives

In the present pilot study, we used a multidisciplinary approach combining molecular-genetic and geolocation data to characterize the chestnut trees of Campania. The resilience and resistance capacity of chestnut forests to environmental factors depends on the specific genotypes present on the territory. Traditional cultivars are a major source of biodiversity; therefore, open and controlled access to chestnut germplasm should be one of the pillars of sustainable agricultural development. Considering that the native chestnut germplasm is of paramount importance for public and private sectors, the possibility that stakeholders have available maps of the genetic distribution of local cultivars will provide them with new potent tools for the management and conservation of these genetic resources. To this end, extensive molecular characterization and geo-spatial cataloging of the chestnut resources in traditionally vocated areas is desirable, in order to activate targeted protection and decision-making processes for effective and efficient use of the chestnut heritage.

## Figures and Tables

**Table 1:** Sampling of 17 individual trees from eight renowned *C. sativa* cultivars in the Regional Park “Volcanic Area of Roccamonfina and Foce Garigliano”, Campania (Italy).

N.	Cultivar	Acronym/Clone	Latitude	Longitude
1	Lucente	LCN1	41.27452	13.97000
2	Marzatica	MRZ1	41.29542	14.01231
		MRZ2	41.29499	14.01185
3	Mercogliana	MRC1	41.27535	13.97022
		MRC2	41.27522	13.97074
4	Napoletana	NPL1	41.27527	13.97010
		NPL2	41.27519	13.97022
		NPL3	41.27509	13.97039
5	Olefarella	OLF1	41.28726	13.99205
6	Paccuta	PCT1	41.31232	13.97400
		PCT2	41.31192	13.97379
		PCT3	41.31189	13.97400
7	San Pietro	SPT1	41.29528	14.01213
		SPT2	41.29548	14.01237
8	Tempestiva	TMP1	41.28671	13.97759
		TMP2	41.28683	13.97763
		TMP3	41.28674	13.97751

## Data Availability

Not applicable.
